# Traumatic Bereavements: Rebalancing the Relationship to the Deceased and the Death Story Using the Two-Track Model of Bereavement

**DOI:** 10.3389/fpsyt.2020.537596

**Published:** 2020-09-15

**Authors:** Simon Shimshon Rubin, Ruth Malkinson, Eliezer Witztum

**Affiliations:** ^1^ International Laboratory for the Study of Loss, Bereavement and Human Resilience, University of Haifa, Haifa, Israel; ^2^ School of Psychological Sciences, University of Haifa, Haifa, Israel; ^3^ Zramim, Postgraduate Psychotherapy Program, University of Haifa, Haifa, Israel; ^4^ Department of Psychology, Max Stern Emek Jezreel College, Emek, Israel; ^5^ Mitra–Israel Center for REBT, Rehovot, Israel

**Keywords:** bereavement, prolonged grief disorder, traumatic bereavement, Two-Track Model of Bereavement, posttraumatic stress disorder, continuing bonds, suicide, psychotherapy

## Abstract

Bereavements that occur under external traumatic circumstances increase the risk for dysfunction, trauma symptomatology, as well as disordered and prolonged grief. While the majority of individuals who have experienced traumatic bereavements do not meet formal criteria for posttraumatic stress disorder (PTSD), persistent complex bereavement disorder (PCBD), or prolonged grief disorder (PGD), the degree of distress and dysfunction for these bereaved can be quite significant. The assessment and intervention paradigms in use with traumatic bereavements often prioritize the trauma and bypass the centrality of the interpersonal loss. By using a bifocal approach in conceptualizing bereavement, the Two-Track Model of Bereavement (TTMB) rebalances the approach to the class of traumatic bereavements. Track I examines biopsychosocial functioning and symptoms of trauma, and track II focuses on the nature of the ongoing relationship with the deceased and the death story that may also have elements of traumatic response. The model and its application serve to identify both adaptive and maladaptive responses to loss along both axes to optimally focus interventions where needed. The story of the death, the psychological relationship with the deceased, and the presence of biopsychosocial difficulties each have a part to play in assessment and intervention. A case study of assessment and intervention following traumatic bereavement due to suicide illustrates how attention to each of these factors in the TTMB can facilitate change. Ultimately, the relational bond with the deceased is a major vector in grief and mourning. Assessment and intervention with traumatic bereavements require attention to dysfunction and symptoms of trauma as well as to the death story and the state of the relationship to the deceased.

## Introduction

Bereavement following the death of a loved one is universal. How one grieves and mourns that loss, and how a broad array of variables influence grief and its outcome have received increasing attention and specification. Since the beginning of the 20^th^ century, the field of thanatology has undergone tectonic changes in the way bereavement is understood. One revolution in bereavement is the break with Freud’s (1) view of mourning as a process leading to the minimization of the emotional connection to the deceased. In place of a view that breaking the bonds (decathexis) with the deceased is adaptive, grieving is recognized as the reworking and maintenance of the attachment bond rather than its severance and has been designated the *continuing bonds* paradigm (2–4). The psychological processes in grief and mourning are related to the life-change reorganization that requires adaptation to the absence of the deceased’s physical presence (5–7). As a life stressor of major proportions, the death of a significant attachment figure puts the bereaved at risk of developing a broad variety of difficulties and dysfunctions including, but not limited to, depressive disorders. At the same time, the majority of bereaved respond to loss without sustained difficulties (8).

The estimated prevalence of bereaved at risk to develop variations of prolonged grief disorder (PGD) and complications of grief generally range between 7-15% across all categories with traumatic circumstances of loss yielding significantly higher estimates (9–13).

## Traumatic Bereavements 

The term *traumatic bereavements* originated in response to clinical reality where bereavements occurring under traumatic circumstances resulted in the familiar mixture of symptom pattern of PTSD together with acute grief (14). Traumatic bereavements increase the risk for dysfunction and symptomatic difficulties and complicated grief (7, 15–18). Traumatic bereavement is a term that stresses the interface of traumatic circumstances with bereavement (14, 19, 20). The potential significance of the interplay between the traumatic and bereavement elements in a variety of loss has received significant attention in the literature over the years, but consensus on diagnosis, research, and intervention remain elusive (7, 20–22).

In a meta-analysis studying the efficacy of grief interventions for a variety of bereavements, both traumatic loss and child loss demonstrated benefits from intervention while “uncomplicated losses” did not show benefit (23). This linkage would support the understanding that both trauma and the death of a child pose extraordinary challenges for post-death grieving and adaptation. Child loss has itself been described as a traumatic loss (24, 25). In our own studies of heightened grief scores with the two-track bereavement questionnaire for complicated grief years after the loss, we noted that the elevated scores that characterized only 5% of adults bereaved of their parents, applied to 10% of the spousal bereaved and to fully 25% of those who had lost children (26). These results support a position that the loss of a child is a loss of traumatic proportions in comparison with other family losses.

The interplay of trauma and bereavement is complex. In research examining adults who were not physically present but were bereaved in the 9-11 attacks, approximately 3 years later 43% received a classification of complicated grief with PTSD among the major comorbid conditions (27). A recent study looked at the presence of symptoms of prolonged grief disorder, posttraumatic stress disorder, and depression for 458 persons presenting for treatment at a Dutch clinic specializing in trauma (28). In the bereaved group, 45% reported a history of violent loss. For their bereaved sample, the authors report clinically relevant PGD scores for 28%, PTSD for 78% and depression for 28%. This overlap between symptoms of trauma and of grief in a clinical setting focusing on trauma is a repeated and robust finding. It underscores the overlap between the two classes of response.

In the framework of the revisions and discussions preceding the DSM -5 (29–31), suggestions to include complicated grief and prolonged grief were rejected. Instead, a new term was added and specified in the category of conditions for further study–persistent complex bereavement disorder (32). This term was put forth to replace terms in use such as pathological grief and complicated grief and was structured with proposed criteria ordered in the current DSM format. It is particularly significant to note that following specified criteria, the diagnostician is requested to address whether the circumstances of death are traumatic:

*Specify if:*

*“With traumatic bereavement: Bereavement due to homicide or suicide with persistent distressing preoccupations regarding the traumatic nature of the death (often in response to loss reminders), including the deceased’s last moments, degree of suffering and mutilating injury, or the malicious or intentional nature of the death”*. [(32), pp.789–792]

The parallel diagnostic formulation for complications of grief in the ICD 11 is classified for the first time under - 6B42 prolonged grief disorder (33). This emphasizes the criteria extended time and dysfunction such that “The disturbance causes significant impairment in personal, family, social, educational, occupational, or other important areas of functioning” (33, 34). In contrast to the DSM-5, the ICD-11 excludes the circumstances of traumatic bereavement from its definition thus ignoring significant component of traumatic bereavement (33). **Table 1** presents the diagnostic criteria for PCBD and PGD. It is important to note that proposed changes for a revised DSM-5 currently under consideration include creating a formal diagnosis of PGD in the DSM section II (35). We agree with those advocating for the inclusion of PGD in a DSM-5 revision in the chapter on trauma and stressor-related disorders rather than in the chapter on depressive disorders (36). Irrespective of any decision on where to place the bereavement diagnosis, we believe it valuable for any modification of the DSM regarding disordered bereavement to retain the specification “with traumatic bereavement” as an important qualifier. The significance of the intersection of trauma and bereavement are consistent findings in the clinical and research literatures (e.g., 35).

**Table 1:** A comparison of the PCBD diagnosis in DSM-5 (2013) and the PGD diagnosis of ICD-11 (2019).

Category	DSM-5 persistent complex bereavement-related disorder (PCBD– conditions for further study)	ICD PGD (2019)
Event	Death of a close other	Death of a close other
Time frame and degree	12 months; “on most days to a clinically significant degree”	6 months and clearly exceeds norms for culture/context
Response focus	1 of yearning/longing; sorrow/pain in response to death; preoccupation with deceased or circumstances of death.	Longing for the deceased or persistent preoccupation with the deceased
Symptoms	6 of the following: 1) marked difficulty accepting the death; 2) disbelief or emotional numbness over the loss; 3) difficulty with positive reminiscing about deceased; 4) bitterness or anger related to the loss; 5) maladaptive appraisals about oneself in relation to deceased or death; 6) excessive avoidance of reminders of the loss; 7) desire to die to be with deceased; 8) difficulty trusting other people since the death; 9) feeling alone or detached from other people since the death; 10) feeling that life is meaningless or empty without deceased or belief that one cannot function without deceased; 11) confusion about one’s role or diminished identity; 12) difficulty pursue interests or plan for future since loss	Persistent and pervasive grief response characterized by longing for the deceased or persistent preoccupation with the deceased accompanied by intense emotional pain (e.g., sadness, guilt, anger, denial, blame, difficulty accepting the death, feeling one has lost a part of one’s self, an inability to experience positive mood, emotional numbness, difficulty in engaging with social, or other activities).
Traumatic death circumstance	Specify if: With traumatic bereavement with persistent distressing preoccupations regarding the traumatic nature of death	No specifier
Degree of impairment	Clinically significant in social, occupational or other important areas of functioning	Significant impairment in personal, family, social, educational, occupational, or other important functioning
Qualifier	Beyond expected norms for relevant cultural, religious, or developmental stage	Exceeds social, cultural, or religious norms

It is premature to determine the extent to which the changes in nomenclature of the DSM and the ICD will impact the ways in which practitioners and researchers approach trauma and bereavement (37, 38). In our experience in Israel and internationally, the emphasis on trauma and PTSD in the context of bereavement has the paradoxical effect of obscuring the centrality of the interpersonal relationship bond in these circumstances (39, 40). One might say that from the trauma perspective, bereavement under traumatic circumstances that results in the familiar symptom pattern of PTSD is PTSD. From the bereavement perspective, however, the symptom pattern of PTSD following the death of a significant relational figure is but one aspect of what may actually be predominantly dysfunctional grief and disordered mourning (41, 42). Furthermore, we believe that there are additional aspects of traumatizing elements that operate in traumatic bereavement. In some cases, the circumstances of the traumatic death events are highly intermeshed with the interpersonal relationship to the deceased that itself has strong conflictual and negative characteristics (17). Bereavements following troubled interpersonal relationships are one example of this. While the DSM-5 describes both homicide and suicide as qualifying as traumatic circumstances, in reality there are important distinctions to be made. One major difference relates to the fact that the person who dies from suicide is both the one who took life and the one whose life was taken. For many of the bereaved, this combination complicates the grief. Overall, assessment and intervention benefit from attention to greater specificity regarding the complicating and traumatizing features involved in bereavement (43).

The literature on trauma and post-trauma has changed how both clinicians and the lay public think about life-threatening events and their impact (32, 44). With the passage of time and the expanding literature base, clinical practitioners and the general public are better informed as to the incidence, prevalence, and pernicious deleterious effects of exposure to traumatic events (45, 46). Similarly, awareness of the various intervention programs and their reported efficacy has increased as well (6, 47, 48).

Alongside the generally positive effect of the expanding wellspring of knowledge and expertise that has accrued to date in the trauma field, there is a less welcome side effect to this phenomenon as it relates to bereavement. Specifically, since major life threatening events directed either at the self or at a loved one are considered events of significant magnitude as to satisfy the criterion for a traumatic stressor, this categorization is often seen to encompass the death of a loved one. Once the death of a loved one is categorized as a major stressor of potentially traumatic proportions, the interpersonal and attachment issues involved in bereavement may be downplayed or missed (41, 42, 49). For example, combat soldiers whose comrades have fallen in battle report attention and follow-up related to the post-trauma and minimal consideration of the grief and mourning for fallen comrades (50).

We shall return to amplify these points with clinical material after the presentation of the Two-Track Model of Bereavement (TTMB).

## Two-Track Model of Bereavement (TTMB): A Model for Research and Practice

The TTMB was developed in order to make sense of research and clinical data that demonstrated successful adaptation to life post loss while maintaining a strong connection to the relationship with the deceased in the present (51, 52). The model itself addresses response to interpersonal loss from a bifocal perspective considering both the biopsychosocial functioning of the bereaved (track I), and the nature of the ongoing relational bond to the deceased (track II), across the life cycle (51, 52). Track I’s focus on biopsychosocial dysfunction reflects both the medico-psychiatric attention to human suffering and the psychological perspectives rooted in the broad domain of mental health, stress, and trauma literatures. Included here are the biological, behavioral, cognitive, emotional, intrapersonal, and interpersonal ways of one’s being in the world. These can be negatively affected following loss for extended periods (5, 14). In cases where there is reason to suspect manifestations of post-trauma, inquiry into the triad of re-experiencing, avoiding, and hyper-alertness is warranted a as part of the assessment (7). Viewed most broadly, the exploration of negative and positive changes in the general domain of function span a wide range of areas. The biospsychosocial approach shares much with the general clinical evaluation of persons facing stressful challenges to their previous mode of living in the world and the way in which they made sense of its meaning (53). These challenges are not fundamentally unique to the bereavement experience and the stresses it brings with it. Thus, exposure to natural disasters, combat, sexual victimization, terror, and a variety of life-threatening situations would warrant the explorations of this first domain in many of the same ways as with the assessment of responses post-bereavement.

**Figure 1:**
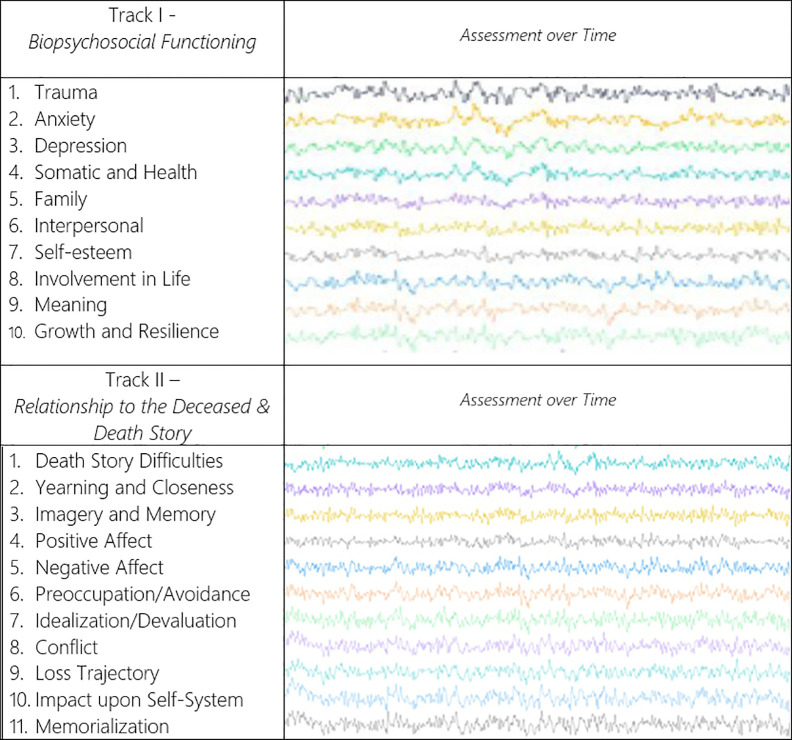
Illustration of the two-track model of bereavement (TTMB)^1^.

The second track of the model, the relationship to the deceased, traces its origins and significance to a wholly different set of assumptions and postulates. In modern terminology, we would stress the relevance of the attachment and object relations literature and the nature of the attachment bond as central to what is unique to bereavement and loss (2, 5, 54). The core insight related to track II of this model is that reworking the relationship to the deceased and the continuing bond with the deceased post-death is a critical feature for understanding response to bereavement. This is the central feature of what makes the loss process unique. The second domain of the two-track framework prioritizes the nature of the relationship to the deceased and the current status of the emotional bond to the deceased. How the psychological experience of the relationship and its accessibility have changed following death are central pieces of this approach (4). An additional focus of track II is rooted in the story of the death and how it has been integrated and assimilated by the bereaved. Particularly in cases of traumatic deaths, the events surrounding the death may remain unassimilated with potentially serious consequences (10, 22). In some cases, this unintegrated and traumatic segment interferes with the processing of the relationship and the access to the broad range of memories, associations, and emotions concerning the life course with the deceased. **Figure 1** illustrates the domains of the TTMB as they unfold over time.

**Figure 2:**
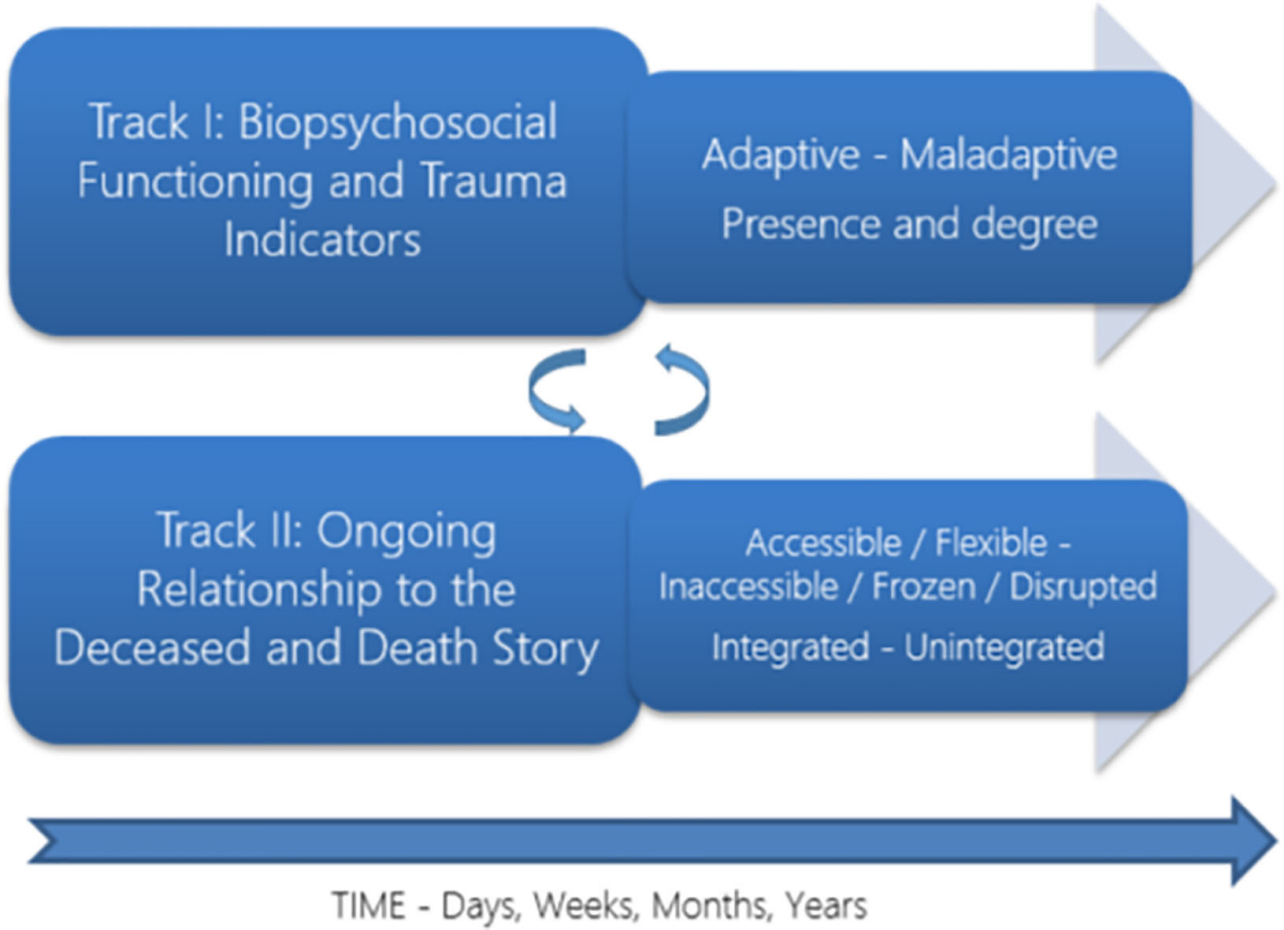
The TTMB in traumatic bereavement^2^.

The importance of combining the two perspectives of functioning and relationship in broad ways formed the basis for the TTMB (7, 51, 52). In this model, the process of adaptation to interpersonal loss is linked to the disruption of homeostatic functioning that accompanies major life stressors on the one hand but also as a byproduct of relating and reconfiguring aspects of the relational bond and attachment system with the deceased on the other hand. The TTMB facilitates the assessment of both functioning and the nature of the continuing attachment to the deceased when significant others die—and this across the entire course of the bereaved person’s lifetime. **Figure 2** conveys the mix of independence and interdependence of the two tracks.

The clinical implications of the model derive directly from its binocular focus. The extent to which potential psychological interventions should privilege one or both domains of the response to loss remains an important clinical question (26). The basic elements of the assessment schema of the TTMB and a rubric for clinician ratings are presented in **Table 2**. The 10 domains of track I’s biopsychosocial functioning and track II’s 11 domains beginning with the death story are relevant in assessing the response to loss at any point in time following the loss. **Table 2** summarizes the scoring scale for clinician assessment of bereaved individuals.

**Table 2:** The TTMB clinician rating scale.

Track I: Biopsychosocial difficulties in functioning and post-trauma	Clinician evaluation		Track II: Ongoing relationship to the deceased and death story			Clinician evaluation	
1. Traumatic responses			1. Death story difficulties/unintegrated/interferes with connection to deceased				
2. Anxious affect and cognitions			2. Yearning and closeness				
3. Depressive affect and cognitions			3. Imagery/memory of deceased				
4. Somatic and Health			4. Positive affect				
5. Familial relationships			5. Negative/unmodulated affect				
6. General interpersonal			6. Preoccupation/avoidance (specify)				
7. Lowered self-esteem and distress			7. Idealization/devaluation (specify)				
8. Involvement in life			8. Conflict in the relationship				
9. Meaning in life			9. Loss trajectory (rate each: shock, searching, disorganization, and reorganization)				
10. Strengths (growth/resilience)			10. Self-system difficulties vis-à-vis deceased and/or death story (specify)				
			11. Memorialization				
**Clinician scoring key**						
Absent/mild/limited	Moderate	Severe/strong		Continue to monitor	Insufficient information	

In assessing the impact of loss in cases of traumatic bereavements, the objective circumstances of the loss, with attendant symptomology characteristic of post-trauma are part of the biopsychosocial response. At the same time, however, inquiry and assessment of the death story are highly significant in assessing how the ongoing relationship to the deceased is affected by that (55, 56). Thus parallel focus is also relevant in relationships where the losses are not death but involve significant changes in requiring adjustment due to major changes in the attachment figure (25, 57). For example, in a recent publication, the rationale for a two-track model of dementia grief (TTM-DG) showed how the particular circumstances involved in caring/caretaking for a close family member suffering from dementia was best understood from the bifocal perspective of the TTMB (58). In particular, the ongoing loss of personhood and identity for a person suffering from cognitive decline and dementia is a challenge and loss for those closest to him or her. For a fuller elaboration of the current applications of the model in clinical work and research, see *Working With the Bereaved: Multiple Lenses on Loss and Mourning* (7).

Focusing on the extent of behavioral difficulties and symptoms of various types is valuable and is sensitive to the ways that bereavement challenges the bereaved to readjust to a new external reality. Whether or not the bereavement response meets criteria for a formal diagnosis, however, many persons seeking assistance after the death of a close person can benefit from the bifocal approach advocated. Many aspects of the relationship to the deceased both pre and post loss require us to learn more about whom was lost. This includes attention to what aspects of that relationship were lost for the bereaved and what portion of that relationship remains active and available to the bereaved after the death. In the case of circumstances of traumatic bereavement, the linkage of trauma with bereavement has often had the paradoxical effect of obscuring the decidedly interpersonal and intrapersonal impact of the loss and replacing it with a focus on the trauma elements (42, 59).

## Working Clinically With the TTMB in a Case of Traumatic Bereavement

The following brief case study of assessment and intervention illustrate the use of the TTMB in a case of traumatic bereavement. In particular, the clinical example illustrates how bereavement can operate as a complex series of traumatizing experiences that have roots and links to both tracks of the TTMB.

Helen, a 36 year old woman, came for treatment to SSR 2 years after the suicide of her husband David. In the initial session, Helen said she was only now ready to get help. Early in the first session, she spontaneously described how she had discovered her husband’s suicide and body. Returning home from a brief business trip abroad, she had opened the door to the master bathroom where she was assaulted by “horrific sights and smells and waves of nausea.” Her husband’s decaying body was lying in the bathtub several days after he had killed himself. He had slit his wrists and the blood flow into the water had left everything a discolored pink after the water had drained away. Her affect as she described her experiences conveyed minimal emotion. While it was clear that the experiences were distressing and painful, her reporting was done in a factual way that was devoid of emotion.

Helen: “Years later, I close my eyes, [and sometimes still] see his body lying there and the red color of the bathtub surrounding him. I have dreams of being physically tortured by someone wearing his face. I have intrusive thoughts about him, this used to be maybe 100 times a day, but now it is much much less. I did most of my work from home and barely went out. I sat in the dark, self-medicated with alcohol and marijuana. I wanted to suffer so I did not see any mental health professionals”.

In the initial meetings, Helen shared her personal history and details about her husband and their time together. She was the eldest of two daughters who grew up in an intact family. She had always been “a bit of a tomboy, interested in sports, computers, and mathematics.” Previous losses included the death of her father when she was 19; the departure of her younger sister for a job overseas, and her mother’s subsequent remarriage and move to the sister’s city of residence. Helen had many strengths and was successful during her years of education, and eventually took her skills set to work in the computer world of high-tech which required a fair amount of international travel. Helen had been introduced to her future husband by a mutual friend and they immediately forged a connection. Soon they were living together, and they married a year later. All told, they had been together for 12 years before David’s suicide. David had been raised in a small town, had done well in school and college, eventually entering into government service. At age 30 he had begun to suffer from depressive episodes. He was seen by a mental health professional and prescribed medications. He stopped both soon thereafter. The couple had weathered several of David’s depressions including one for which he was hospitalized briefly. Several weeks before her business trip abroad, David had again gone into what seemed to be a depressive mood, but he assured her that his depression was under control and there was no need to see anyone. He said he would be fine so she need not be concerned for his wellbeing. During her stay overseas, they were in contact for the first 2 days before he told her that he was going to be incommunicado for several days as he was going to hike in an area where there was no cellphone coverage. Upon her return, she found his lifeless body at home.

At the time of her request for therapy, Helen exhibited some symptoms of stress and depressive affect, but these did not significantly interfere significantly with her day to day functioning. She had seen a physician for medication for sleep difficulties the first year, but was now sleeping relatively well although there were the occasional nightmares reported. As indicated above, she continued to have intrusive thoughts and images of the death scene on a daily basis, but these were no longer interfering with her functioning. She continued to avoid places that reminded her of David’s depressions and she had withdrawn from interactions with their mutual friends. She described heightened vigilance and anxiety in response to phone calls. Not infrequently, and particularly when the phone rang at odd hours, she felt a reflexive fear that the call was about bad news affecting her family overseas. These responses were much reduced from what she had experienced for many months following David’s suicide.

Queried about the nature of the memories, thoughts and feelings about the relationship with David, Helen said that she felt shut down and unable to connect with their good times together. She felt guilty for having left him to go on her business trip, denied feelings of anger vis-a-vis the suicide or his deception and the way he hid his state of mind from her. The memories of David and the recollections of the times when she had enjoyed his company eluded her along with the feelings of pleasure they once held.

### Assessment and Intervention Plan With the TTMB

The diagnostic categories applicable to Helen in the DSM-5 were from the trauma and stressor related disorders (32). Helen’s current functioning involved significant suffering and many symptoms associated with post-trauma although not sufficiently meeting criteria for a DSM-5 diagnosis of PTSD. The experience of her husband’s suicide met the criterion for exposure to a traumatic event; there were intrusions of the death scene; avoidance of places that triggered memories; negative alterations in mood, and a degree of hyper alertness as manifest in her response to phone ringing. The circumstances and degree of clinically significant distress met conditions for “Other Specified Trauma- and Stressor-Related Disorder” with the further specification of persistent complex bereavement disorder. We would point out that this mix of trauma and bereavement in the more formal diagnosis reflects clinical, conceptual and diagnostic overlap, and interpenetration.

From the perspective of the TTMB, Helen had significant distress on many of the track I biopsychosocial categories. Feelings of anxiety, depression, a sense that her life was adrift and without meaning, reduced involvement in social interactions with family and friends, along with her lowered self-esteem reflected the extent to which her biopsychosocial functioning was impaired. At present, on the trauma dimension, there were elements of the triad of intrusions/re-experiencing, avoidance, and hyperarousal. These were significant and consistent with a residue of active post-trauma.

Examining track II’s death story and relational bond with her deceased husband conveyed significant difficulties. In the quote included earlier, Helen graphically described David’s suicide and the experience of finding his body. The story of the death emphasized the shocking elements, but nowhere was she able to integrate this experience into a broader narrative. There were no indications of some degree of acceptance and assimilation of this experience with the bulk of her relationship with David. Helen returned repeatedly to the question of why he had killed himself in the way that he did without caring about what it would be like for her to find him like that. The narrative of his death did not fit into her description of his depressions and struggles with suicidal thoughts. It was as if this experience began and stopped with the discovery of his body days after his death.

The story of the relationship with David and the limited degree to which memories of their lives together were accessible to her indicated that the relationship bond required therapeutic attention. Helen had difficulty accessing the wellspring of memories of their relationship. Memories of the positive elements of their relationship were few and vague. There was little in the way of perspective or the ability to reference the thick history of their years together. Negative and conflictual elements of the connection with David were present and associated with pain. She felt guilty over having left David to go overseas and also a sense of general unworthiness and failure as a spouse. Despite the objective dislocation that David’s death had caused her, she repeated stated that she was unable to be angry at him for what he had done. With access to positive memory and emotions of their relationship constricted and with a painful focus on David’s suicide in the fore, the relationship domain was problematic. In this condition, progression toward adaptive grief and mourning were stymied (2, 5, 7).

Helen was having difficulties on track I and track II and the question of how to focus intervention with respect to these variables was highly relevant. On track I, the elements of trauma, depressive thoughts and emotions, lowered self-esteem, constricted connection with life tasks, connecting to meaning in life, were all rated by SSR as elevated and of moderate severity. The combination of these variables, together with limited family support and other interpersonal relationships, powerfully affected Helen’s ability to engage in her life. These track I biopsychosocial difficulties convey significant dysfunction and distress and intervention would need to take them into account.

On track II, Helen had not integrated the death story nor had she been able to retell the story with any degree of flexibility or perspective. The ongoing sense of shock in the way the death event was experienced interfered strongly with her ability to fully describe her husband and the many years of their relationship. This aspect of the death-related trauma is rated on track II because of its significance in impeding grief and mourning and for its interference with access to the interpersonal relationship and the continuing bond with the deceased. The relationship with David was scored as highly significant with the variables related to the death story, negative affect, preoccupation with the deceased, conflict in the relationship, and assault to the self-system as a result of the relationship being coded as of severe proportions. Yearning was pronounced, while positive affect, memories of the good times, and progress toward memorialization of the deceased were limited. **Table 3** gives the clinician scoring of Helen at the end of the intake evaluations on the TTMB variables discussed.

**Table 3:** Helen–Clinical Assessment with the TTMB rating scale at beginning of treatment.

Track I: Biopsychosocial functioning	Clinician evaluation		Track II: Relationship to the deceased and death story		Clinician evaluation	
1. Traumatic responses	Moderate		1. Death story		Severe	
2. Anxious affect/cognitions	Mild		2. Yearning and closeness		Moderate	
3. Depressive affect/cognitions	Moderate		3. Imagery/memory		Limited	
4. Somatic and health	Mild		4. Positive Affect		Limited	
5. Familial relationships	Mild/limited		5. Negative Affect		Severe	
6. General interpersonal	Mild/limited		6. Preoccupation/Avoidance		Severe	
7. Lowered self-esteem	Moderate		7. Idealization/devaluation		Mild–deval	
8. Involvement in life	Moderate		8. Conflict in the relationship		Severe	
9. Meaning in life	Moderate		9. Loss Trajectory (Shock, Searching, Disorganization, and Reorganization)		Sh- Severe Se – Limited Dis- Moderate ReO - Limited	
10. Strengths (growth/resilience)	Limited		10. Self-system		Severe	
			11. Memorialization		Limited	
**Clinician scoring key**						
Absent/mild/limited	Moderate	Severe/strong		Continue to monitor		Insufficient information

### The Treatment Plan Based on the TTMB

In considering a treatment plan, once again, the TTMB framework was used in formulating the intervention plan. Helen’s response to the traumatic loss 2 years post-death was characterized by significant difficulties in functioning, features of traumatic stress, and relation based elements that served to reinforce each other. They limited her ability to function and her ability to grieve in ways that facilitated working through of aspects of the loss. With difficulties on both tracks, it was important to formulate an intervention plan that would take into account both domains of the TTMB.

The focus on track I’s biopsychosocial functioning and trauma suggested a range of intervention possibilities. The treatment components most relevant for the biopsychosocial difficulties combined elements of supportive psychotherapy with a variety of techniques and exercises that have been associated that included techniques drawn from evidence based treatment strategies (60, 61).

**Figure 3:**
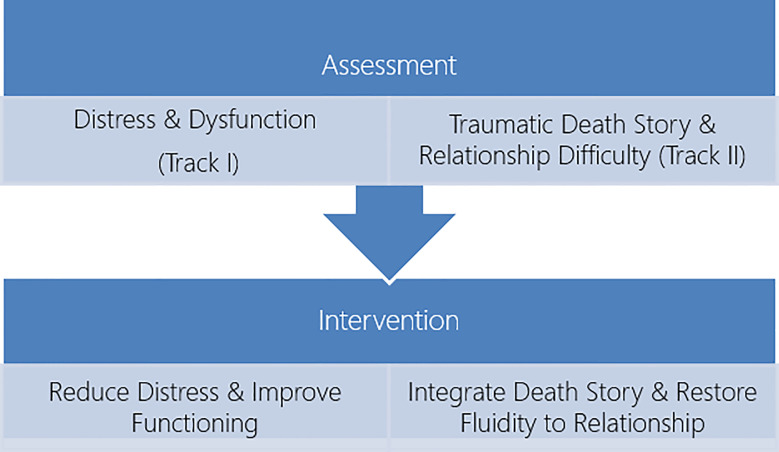
Overview of the assessment and intervention plan with Helen.

Concurrent with interventions related to track I, the focus on track II drew attention to the importance of transforming the death story. The frozen and shocking death scene effectively locked the death by suicide event into a kind of video clip that did not develop into a story. Finding ways to help Helen take the death story and continue on beyond the death scene, ultimately integrating it to the broader story of David’s life, death, and memory were an overarching goal of the track II intervention. The goals of treatment here emerged squarely from the track II conceptualization of the significance of the continuing bond to, and the dynamic relationship with, the complex of memories, thoughts and emotions linked to the deceased. This connection can serve as a positive and supportive presence for the bereaved across the life span (7). **Figure 3** illustrates the double focus of the intervention plan and goals.

Whatever the foci and plans for therapy, it was important to get Helen’s cooperation and to enlist her in the treatment plan. Following the initial sessions of history taking, the therapist shared the basic outline of the TTMB as a way of thinking about responses to loss with the goal of psychoeducation and helping Helen make sense of her response to David’s suicide. Following a discussion of the model, the predominant areas of difficulty and dysfunction that she experienced were shared back with her. Therapist and patient were in agreement on the need for intervention, and the areas in her life that were particularly troubling. The goals of treatment were to help her feel better, to assist her with the various domains of functioning that troubled here, to ease the pain and shock of her memory of David’s death, and to help her regain a more flexible and supportive connection with his memory.

### Critical Junctures in Helen’s Treatment

The initial weeks of treatment were devoted to building the therapeutic alliance with Helen. In this initial stage of therapy she filled in details of her current life and the nature of the challenges she faced as well as her feelings of estrangement and longing for David. In order to assist her in reducing her anxiety, depression, and tension, one set of interventions were aimed at increasing her ability to modulate her affect. Self-observation, journaling, and release of tension *via* physical exercise were prescribed, and she was able to follow through on all these (7). The benefits of mindfulness were explained, and practice in the sessions was introduced (62). Even though she did not continue with mindfulness, the focus on allowing emotions and thoughts to be seen as reflecting the ebb and flow of the mind rather than as thoughts to engage or avoid were helpful to her. Above all, encouraging her to share her thoughts and feelings about those encounters and areas of her life that troubled her without the requirement that they reflect the trauma or the loss were beneficial. This allowed her to explore her thoughts regarding meaning in life, her mixed feelings toward family and friends, and the nature of difficulties in her work and home that were of benefit to her. As she followed through with regular exercise, sporadic periods of mindfulness at home, sharing her thoughts in the sessions and journaling occasionally at home, her mood and sense of self began to improve. These in turn contributed to the strengthening of the therapeutic alliance.

The next stage of treatment began after the therapeutic alliance and initial benefits from treatment were in place. Building on the therapeutic alliance, the major intervention focus directed at Helen’s relationship to David and the manner of his death became the center of this next phase of treatment.

The techniques used in psychotherapy for bereavement are myriad (60). In situations where the relationship is constrained or moribund finding ways to open possibilities for change and flow in the relationship can be transformative. Chair work where the bereaved and deceased “interact” and letter writing to the deceased are among the better known of these techniques (60, 63). From the TTMB perspective, in the case of Helen, because the circumstances of her husband’s death were so traumatic, they effectively stymied her ability to reorganize her relationship to him. The therapeutic technique was designed to permit Helen to soften the boundaries between the living and the dead, the real and the imaginary, and to introduce the possibility of change and the evolution of the relationship. One could think of this intervention technique as a way of seeking to open “transitional space and fluidity” into what had been a frozen and “dead” relationship (64). At the same time, this intervention technique had potential to allow for the evolution in the story of David’s death and the discovery of his body that had heretofore remained traumatic, stuck, and repetitious.

Some 3 months into the therapy, having made gains in emotion regulation, having taken up walking for an hour each day, and having attempted mindfulness but deferred it for later, the more direct focus on Track II’s relationship began. The letter writing that involved having Helen write a letter to David with the goal of facilitating the reconnection and expansion of the current relationship. (See chapter 10, pp 151–171 in (7) for a fuller description of this type of intervention.)

The first letter she composed was straightforward:

Dear David,

There are so many things I want to say to you…. things I’ve thought these past several years…. I want to be angry at you for what you did, what you promised me you would never do. I thought you were getting better, …

You promised you wouldn’t hurt yourself, because you said you didn’t want to hurt me. So why did you do it? …. And to do it like that, for me to find you? … how could you never consider what it would do to me, to find you like that?

I said I want to be angry at you, and as much as I want answers to these questions the truth is I can’t be angry with you I just miss you so much. All I think about is what I would do or give up to have another 5 minutes together, just to talk to you and hug you.

In this first letter, the connection is made, and the longing and wish for reconnection are present. She introduces the possibility of complaints directed toward David for his act of suicide, but anger and disappointment are mentioned though not experienced. The longing for the reconnection are very much present.

The next letter was written two weeks later

Dear David,

Starting these letters is always so difficult. It feels strange, like I’m speaking to you again back when you were alive. … And yet this also feels more impersonal, as even though I feel I am talking to you, I feel less close to you then I did in the past. Maybe that is because I am angry over what you did. … The truth is that I don’t know what has caused it but my feelings for you have changed, and though I think of you constantly still it is not through the same rose colored glasses I once did.

I find myself feeling frustrated more and more by the fallout of your suicide. For so long I blamed you … How could you not consider what would happened to me, how utterly your actions would damage and destroy my life. (2nd letter).

### Commentary

The degree of change between letter one and two is striking. Both letters speak to the ability of Helen to enter into the letter writing exercise and to find herself opening up with her emotions and entering into a dialogue with David. This entrance into a living dialogue brings with it potential for change. The emotion of anger that is denied in letter one emerges strongly in letter 2. More importantly, she indicates that an idealized picture of David referred to in the rose colored glasses of letter two have been replaced by a more sober assessment. The shift in how David is viewed and the change it brings with it in the relationship are pivotal in allowing grief to progress as Helen has more freedom to think about and experience a broader range of emotion than she had earlier. The second letter served as a transformative experience for Helen. Following the letter, Helen spoke about remembering more of her time together with David, and he felt both nearer to her and more alive than she had experienced him for a long time. The broadened access to memories of David, the anger focused on David instead of only on herself, and the story of the depressions that became part of the narrative around the suicide all reflected the movement toward grieving and mourning. Connecting more fully to David also allowed her to become more whole as well.

The sense of betrayal and assault that Helen experienced in response to the suicide were a traumatizing experience for her. Most importantly, the act of suicide served to shatter the person and internal working model of the David she had known. Not only were the memories and connections to him less available, they were also colored by the action that changed the way she viewed him and their relationship. So that in addition to the trauma of the discovery of the dead body and the assault on the senses involved with that, there was an additional trauma, and one that directly related to the experience of who her husband was for her. Helen in effect was dealing with a double trauma, one of external characteristics of the encounter with the death scene, and one that was rooted in the traumatic encounter with her experience of who her husband was for her, and his actions toward her. On the one hand, the Track I trauma has an objective component of exposure to David’s death and his dead body. On the other hand, the Track II trauma was also centered around the relationship with David and the betrayal that his suicide brought to Helen. The death story remained one that she had been unable to integrate and weave into the story of their relationship.

### A Brief Note on the Treatment Conclusion

Helen’s initial letters to David were followed by more letters, by chair work where she took both roles in the dialogue with her deceased husband, and by her newly recovered ability to talk about their relationship. She continued her journaling, exercise, and self-care activities. The reconnection to the relationship with David allowed her to integrate the death story with the relationship in a way that prioritized David’s struggles with depression as most significant, and the mode of death and death scene as secondary. Positive memories, humorous incidents, and discussion of the painful periods of depression came up regularly. Treatment continued for another half-year. Notable changes followed and included a deeper engaged with her work, family, and friends; increasing positive emotions; and reduction in traumatic imagery and dreams. Yearning for David was less pronounced, the death story was better integrated with the life story, and Helen found increased support in the memory of the good times in her relationship with David. In the concluding sessions, her experience of the therapist and therapeutic relationship, and how these contributed to her improvement, were part of the closing of this treatment.

## Concluding Remarks

The interplay between trauma and bereavement in traumatic bereavements has received much attention but consensus remains elusive (65). Attention to the extreme stresses involved in bereavements occurring under traumatic circumstances is critical to understanding both adaptive and maladaptive bereavement responses. At the same time, the decidedly interpersonal aspects of bereavement, grief and mourning should remain squarely in the center of the understanding of traumatic loss. It is a mark of the maturation of the bereavement field that many clinicians with a bereavement focus pay particular attention to circumstances of trauma. The traumas involved in the death circumstances can be joined by traumas related to the psychological representations of the attachment bond and interpersonal relationship. Any number of bereavement circumstances can result in the bereaved responding with a combination of trauma-related elements. These may be most appropriately understood from a combination of the biopsychosocial (including stress and PTSD), the degree of integration of the story of the death, and the degree and types of psychological engagement with the continuing bond with the deceased. We advocate for the use of the TTMB because its conceptual and applied perspective address the relevant biopsychosocial functioning, death narrative, and relationship with the deceased variables (7, 14, 56).

Clinical work is often geared to assessment and interventions seeking to restore balanced biopsychosocial functioning. In interventions following disordered and maladaptive bereavement, the rebalancing and reworking of the relationship to the deceased may proceed without any direct assistance or awareness of the therapist. In other situations, therapy may focus on the ongoing relationship to the deceased without particular attention to the biopsychosocial status of the griever. It is most efficacious, however, for clinicians to use a balanced approach such as that of the TTMB to address both function and the relationship to the deceased. Such an approach facilitates the return to more adequate function, growth, and adaptation to loss over time.

## Author Contributions

SSR, RM, and EW contributed to the design and organization of the article. SSR supplied the case material. RM and EW commented and expanded the discussion of the case. All three are responsible for **Table 1** and **Figures 2** and **3**. SSR provided **Figure 1** and **Tables 2** and **3**. EW spearheaded the consideration of the diagnostic issues in the DSM and the ICD, was in charge of references, and fact checked all material. SSR was the lead author who did the most of the writing and editing. RM and EW expanded and critiqued each draft leading to the final product. The authors view this as a team effort. The order of the names reflects not only the extent of contributions on the one hand but also the three names testify to the fact that “the whole is more than the sum of its parts.”

## Conflict of Interest

The authors declare that the research was conducted in the absence of any commercial or financial relationships that could be construed as a potential conflict of interest.

## Footnotes

1 The assessment over time waves represent pictorially how a series of time points would look if graphed. The amplitudes are for illustration of fluctuation and not for purposes of conveying degree or direction of the variables.

2 Reprinted with permission from Rubin et al., 2012

## References

[1] Freud S Mourning and Melancholia. In: Strachey J, editor. Standard edition of the complete psychological works of Sigmund Freud, vol. 14 . London: Hogarth (1957). p. 237–58. (Original work published 1917).

[2] Klass D Silverman P Nickman S eds. Continuing bonds: New understanding of grief. Taylor & Francis: Washington, DC (1996).

[3] Klass D Steffen E eds. Continuing bonds in bereavement: New directions for research and practice. Routledge: New York (2018).

[4] Rubin SS The resolution of bereavement: A clinical focus on the relationship to the deceased. Psychotherapy (1985) 22(2):231–5. 10.1037/h0085499

[5] Bowlby J “Attachment and loss”. In: Loss, sadness and depression, vol. 3 . New York: Basic Books (1980).

[6] Malkinson R “Cognitive grief therapy: Constructing a rational meaning to life following loss”. In: . PsycNET. New York: W W Norton & Co (2007). Available at: https://psycnet.apa.org/record/2006-13248-000. [cited 2020 Aug 18]. Available from.

[7] Rubin SS Malkinson R Witztum E Working with the bereaved: Multiple lenses on loss and mourning. Routledge: New York (2012).

[8] Bonanno GA Loss, Trauma, and Human Resilience: Have We Underestimated the Human Capacity to Thrive after Extremely Aversive Events? Am Psychol (2004) 59(1):20–8. 10.1037/0003-066X.59.1.20 14736317

[9] Kersting A Brähler E Glaesmer H Wagner B Prevalence of complicated grief in a representative population-based sample. J Affect Disord (2011) 131(1–3):339–43. 10.1016/j.jad.2010.11.032 21216470

[10] Kristensen P Weisaeth L Heir T Bereavement and mental health after sudden and violent losses: A Review. Psychiatry (2012) 75(1):76–97. 10.1521/psyc.2012.75.1.76 22397543

[11] Lundorff M Holmgren H Zachariae R Farver-Vestergaard I O’Connor M Prevalence of prolonged grief disorder in adult bereavement: A systematic review and meta-analysis. J Affect Disord (2017) 212:138–49. 10.1016/j.jad.2017.01.030 28167398

[12] Shear MK Grief and mourning gone awry: Pathway and course of complicated grief. Dialogues Clin Neurosci (2012) 14(2):119–28. 10.31887/DCNS.2012.14.2/mshear PMC3384440 22754284

[13] Steil R Gutermann J Harrison O Starck A Schwartzkopff L Schouler-Ocak M Prevalence of prolonged grief disorder in a sample of female refugees. BMC Psychiatry (2019) 19(1):148. 10.1186/s12888-019-2136-1 31088419 PMC6518607

[14] Malkinson R Rubin SS Witztum E eds. Traumatic and Non-traumatic Loss and Bereavement: Clinical Theory and Practice. International /Psychosocial Press: Madison, CT (2000).

[15] Barlé N Wortman CB Latack JA Traumatic bereavement: Basic research and clinical implications. J Psychother Integr (2017) 27(2):127–39. 10.1037/int0000013

[16] Shear MK Smith-Caroff K Traumatic loss and the syndrome of complicated grief. PTSD Res Q (2002) 13(1):1–16. 10.1037/e400332008-001

[17] Rubin SS Malkinson R Witztum E Clinical aspects of a DSM Complicated Grief Diagnosis: Challenges, dilemmas, and opportunities. In: Stroebe MS Hansson RO Schut H Stroebe W, editors. Handbook of bereavement research and practice: Advances in theory and intervention. Washington, DC: American Psychological Association Press (2008). p. 187–206.

[18] Stroebe MS Hansson RO Schut H Stroebe W eds. Handbook of Bereavement Research and Practice. In: . Advances in Theory and Intervention. Washington, DC: American Psychological Association Press.

[19] Smid GE Kleber RJ de la Rie SM Bos JBA Gersons BPR Boelen PA Brief eclectic psychotherapy for traumatic grief (BEP-TG): Toward integrated treatment of symptoms related to traumatic loss. Eur J Psychotraumatol (2015) 6. 10.3402/ejpt.v6.27324 PMC4495623 26154434

[20] Stroebe M Schut H Finkenauer C The traumatization of grief? A conceptual framework for understanding the trauma-bereavement interface - PubMed. Isr J Psychiatry (2001) 38(3–4):185–201. 11725417

[21] Neria Y Litz BT Bereavement by traumatic means: The complex synergy of trauma and grief. J Loss Trauma (2004) 9(1):73–87. 10.1080/15325020490255322 23633929 PMC3637930

[22] Rynearson EK Retelling violent death. Routledge: New York (2001).

[23] Currier JM Neimeyer RA Berman JS The Effectiveness of Psychotherapeutic Interventions for Bereaved Persons: A Comprehensive Quantitative Review. Psychol Bull (2008) 134(5):648–61. 10.1037/0033-2909.134.5.648 18729566

[24] Christ GH Bonanno G Malkinson R Rubin SS Bereavement experiences after death of a child. Appendix E. In: Field MJ Behrman RE, editors. When children die: Improving palliative and end-of-life for children and their families. Washington, DC: Institute of Medicine National Academy Press (2003).

[25] Yehene E Brezner A Ben-Valid S Golan S Bar-Nadav O Landa J Factors associated with parental grief reaction following pediatric acquired brain injury. Neuropsychol Rehabil (2019) 45(1):1–24. 10.1080/09602011.2019.1668280 31556807

[26] Rubin SS Bar-Nadav O The Two-Track Bereavement Questionnaire for Complicated Grief (TTBQ-CG31). In: Neimeyer RA, editor. Techniques of Grief Therapy: Assessment and Intervantion. New York: Routledge (2016). p. 87–98.

[27] Neria Y Gross R Litz B Maguen S Insel B Seirmarco G Prevalence and psychological correlates of complicated grief among bereaved adults 2.5-3.5 years after september 11th attacks. J Trauma Stress (2007) 20(3):251–62. 10.1002/jts.20223 17597124

[28] Djelantik A Robinaugh DJ Kieber RJ Smid GE Boelen PA Symptomatology following loss and trauma: Latent class and network analyses of prolonged grief disorder, posttraumatic stress disorder, and depression in a treatment seeking trauma-exposed sample. Depress Anxiety (2020) 37(1):26–34. 10.1002/da.22880 30724427 PMC7004006

[29] Prigerson HG Horowitz MJ Jacobs SC Parkes CM Aslan M Goodkin K Prolonged Grief Disorder: Psychometric Validation of Criteria Proposed for DSM-V and ICD-11. Brayne C, editor. PLoS Med (2009) 6(8):e1000121. 10.1371/journal.pmed.1000121 19652695 PMC2711304

[30] Shear MK Simon N Wall M Zisook S Neimeyer R Duan N Complicated grief and related bereavement issues for DSM-5. Depress Anxiety (2011) 28(2):103–17. 10.1002/da.20780 PMC3075805 21284063

[31] Wagner B Maercker A The Diagnosis of Complicated Grief as a Mental Disorder: A Critical Appraisal. Psychol Belg (2010) 50(1–2):27. 10.5334/pb-50-1-2-27

[32] American Psychiatric Association Diagnostic and Statistical Manual of Mental Disorders. 5th Ed American Psychiatric Association Press: Arlington (2013).

[33] World Health Organization International classification of diseases for mortality and morbidity statistics (11th Revision). (2018).

[34] World Health Organization ICD-11 - Mortality and Morbidity Statistics - 6B42 Prolonged Grief disorder. (2019). Available at: https://icd.who.int/browse11/l-m/en#/http://id.who.int/icd/entity/1183832314.

[35] American Psychiatric Association View and Comment on Recently Proposed Changes to DSM–5. (2020). Available at: https://www.psychiatry.org/psychiatrists/practice/dsm/proposed-changes.

[36] Eisma MC Boelen PA Lenferink LIM Prolonged grief disorder following the Coronavirus (COVID-19) pandemic. Psychiatry Res (2020) 288:113031. 10.1016/j.psychres.2020.113031 32360895 PMC7194880

[37] Killikelly C Maercker A Prolonged grief disorder for ICD-11: the primacy of clinical utility and international applicability. Eur J Psychotraumatol (2017) 8(sup6):1476441. 10.1080/20008198.2018.1476441 29887976 PMC5990943

[38] Maciejewski PK Prigerson HG Prolonged, but not complicated, grief is a mental disorder. Br J Psychiatry (2017) 211(4):189–91. 10.1192/bjp.bp.116.196238 28970298

[39] Maercker A Znoj H The younger sibling of PTSD: similarities and differences between complicated grief and posttraumatic stress disorder. Eur J Psychotraumatol (2010) 1(1):5558. 10.3402/ejpt.v1i0.5558 PMC3402016 22893801

[40] Lahav Y Solomon Z From reliving to remembering: Treatments for trauma. Reisling. (Hebrew: Israel (2019).

[41] Witztum E Malkinson R Rubin SS Traumatic grief and bereavement resulting from terrorism: Israeli and American perspectives. In: Heilman SC, editor. Death, bereavement, and mourning. New York: Transaction Books (2005).

[42] Witztum E Malkinson R Rubin SS Loss, traumatic bereavement and mourning culture: The Israel example. In: Ataria Y Gurevitch D Pedya H Neria Y, editors. International Handbook of Trauma and Culture. New York: Springer (2016).

[43] Layne CM Kaplow JB Oosterhoff B Hill RM S. Pynoos R The Interplay between Posttraumatic Stress and Grief Reactions in Traumatically Bereaved Adolescents: When Trauma, Bereavement, and Adolescence Converge. Adolesc Psychiatry (Hilversum) (2017) 7(4):266–85. 10.2174/2210676608666180306162544

[44] Herman J Trauma and recovery. Basic Books: New York (1997).

[45] Eilefson SL The Trauma Thesis: Medical and Literary Representations of Psychological Trauma in the Twentieth Century. Loyola University Chicago: Chicago, IL (2015). Available at: https://ecommons.luc.edu/luc_diss/1635.

[46] Kleber RJ Trauma and Public Mental Health: A Focused Review. Front Psychiatry (2019) 10(JUN):451. 10.3389/fpsyt.2019.00451 31293461 PMC6603306

[47] Malkinson R The ABC of rational response to loss. In: Niemeyer RA, editor. Techniques of grief therapy: Creative principles for counseling the bereaved. New York: Routledge (2013). p. 129–32.

[48] Watkins LE Sprang KR Rothbaum BO Treating PTSD: A Review of Evidence-Based Psychotherapy Interventions. Front Behav Neurosci (2018) 12:258. 10.3389/fnbeh.2018.00258 30450043 PMC6224348

[49] Green BL Traumatic loss: Conceptual and empirical links between trauma and bereavement. J Pers Interpers Loss (2000) 5(1):1–17. 10.1080/10811440008407845

[50] Rubin SS Malkinson R Koren D Mor Yosef S Witztum E Military bereavement and combat trauma. In: Ringel S Brandell JR, editors. Trauma: Contemporary Directions in Theory, Practice, and Research. Thousand Oaks, CA: SAGE Publications (2011).

[51] Rubin S A two-track model of bereavement: Theory and application in research. Am J Orthopsychiatry (1981) 51(1):101–9. 10.1111/j.1939-0025.1981.tb01352.x 7212022

[52] Rubin SS The Two-Track Model of bereavement: Overview, retrospect and prospect. Death Stud (1999) 23(8):681–714. 10.1080/074811899200731 10848088

[53] Neimeyer RA ed. Meaning Reconstruction and the Experience of Loss. American Psychological Association: Washington, DC (2001).

[54] Bar-Nadav O Rubin SS Love and Bereavement: Life Functioning and Relationship to Partner and Spouse in Bereaved and Nonbereaved Young Women. OMEGA - J Death Dying (2016) 74(1):62–79. 10.1177/0030222815598035

[55] Kosminsky PS Jordan JR Attachment-informed grief therapy: The clinician"s guide to foundations and applications. Routledge: New York (2016).

[56] Rubin SS Malkinson E Witztum E The Two-Track Model of Bereavement and Continuing Bonds. In: Klass D Steffen E, editors. Continuing bonds in bereavement: New directions for research and practice, 2nd Ed New York: Routledge (2018). p. 17–30.

[57] Yehene E Zaksh Y Davidian M Bar-Nadav O Elyashiv M Locked-in your heart-shaped box: Familial-role and attachment orientation as predictors of grief in prolonged disorders of consciousness vs. death. Death Stud (2020) 44(8):510–20. 10.1080/07481187.2019.1586795 30938582

[58] Rubin SS Manevich A Doron II The Two-Track Model of Dementia Grief (TTM-DG): The theoretical and clinical significance of the continuing bond in sickness and in death. Death Stud (2019) 1–17. 10.1080/07481187.2019.1688014 31713463

[59] Cloitre M The “one size fits all” approach to trauma treatment: should we be satisfied? Eur J Psychotraumatol (2015) 6(1):27344. 10.3402/ejpt.v6.27344 25994021 PMC4439409

[60] Neimeyer RA Techniques of grief therapy: Creative principles for counseling the bereaved. Niemeyer RA, editor. Routledge: New York (2013).

[61] Lambert MJ ed. Bergin and Garfield"s handbook of psychotherapy and behavior change. 6th Ed Wiley: New York (2013).

[62] Williams M Teasdale J Segal Z Kabat-Zinn J The Mindful Way through Depression. Guilford: New York (2007).

[63] Witztum E van der Hart O Friedman B The use of metaphors in psychotherapy. J Contemp Psychother (1988) 18(4):270–90. 10.1007/BF00946010

[64] Winnicott D Playing and reality. Basic Books: New York (1971).

[65] Djelantik AAAMJ Smid GE Mroz A Kieber RJ Boelen PA The prevalence of prolonged grief disorder in bereaved individuals following unnatural losses: Systematic review and meta regression analysis. J Affect Disord (2020) 265:146–56. 10.1016/j.jad.2020.01.034 32090736

